# Supporting general adult psychiatry higher trainees to develop research competencies: a training improvement project

**DOI:** 10.1192/bjo.2021.377

**Published:** 2021-06-18

**Authors:** Annalie Clark, John Stevens, Sarah Abd El Sayed

**Affiliations:** 1 Greater Manchester Mental Health NHS Foundation Trust; 2 Mersey Care NHS Foundation Trust; 3 Greater Manchester Mental Health NHS Foundation Trust

## Abstract

**Aims:**

Evidence shows that research-active trusts have better clinical patient outcomes. Psychiatric trainees are required to develop knowledge and skills in research techniques and critical appraisal to enable them to practice evidence-based medicine and be research-active clinicians. This project aimed to evaluate and improve the support for developing research competencies available to general adult psychiatry higher trainees (HT) in the North-West of England.

**Method:**

General Adult HT in the North–West of England completed a baseline survey in November 2019 to ascertain trainee's experience of research training provision. The following interventions were implemented to address this feedback:

A trainee research handbook was produced, containing exemplar activies for developing research competencies and available training opportunities, supervisors and active research studies.

The trainee research representative circulated research and training opportunities between November 2019 – August 2020.

Research representatives held a trainee Question and Answer session in September 2020.

All General Adult HT were asked to complete an electronic survey in November 2020 to evaluate the effect of these interventions.

**Result:**

18 General Adult HT completed the baseline survey in November 2019. 29.4% of trainees thought they received enough information on research competencies and 88.9% wanted more written guidance. 38.9% of trainees knew who to contact about research within their NHS Trust and 33.3% were aware of current research studies. Identified challenges for meeting research competencies included lack of time, difficulty identifying a mentor and topic and accessibility of projects.

20 General Adult HT completed the repeat survey in November 2020. 50% of trainees wanted to be actively involved in research and 35% wanted to develop evidence-based medicine skills. A minority of trainees aimed to complete only the minimum ARCP requirements. All trainees thought the handbook was a useful resource for meeting research competencies and would recommend it to other trainees. In trainees who received the handbook, 94.7% thought they had received adequate support on meeting research competencies and 94.7% knew who to contact about research in their trust. 68.4% of trainees would like further written guidance on meeting research competencies. Trainees highlighted ongoing practical difficulties with engaging with research and concern about lacking required skills for research.

**Conclusion:**

Trainees are motivated to engage with research on various different levels, not purely for ARCP purposes. Simple interventions can help trainees feel adequately supported with meeting research competencies. Further work to support trainee involvement in research and improve trainee confidence in engaging with research is required.

